# Transcriptomic Analysis of Long Non-Coding RNA during *Candida albicans* Infection

**DOI:** 10.3390/genes14020251

**Published:** 2023-01-18

**Authors:** Gabriela Flores Gonçalves, Joice de Faria Poloni, Márcio Dorn

**Affiliations:** 1 Center for Biotechnology, Federal University of Rio Grande do Sul, Porto Alegre 91501-970, Brazil; 2 School of Health and Life Sciences, Pontifical Catholic University of Rio Grande do Sul, Porto Alegre 90619-900, Brazil; 3 National Institute of Science and Technology—Forensic Science, Porto Alegre 91501-970, Brazil; 4 Institute of Informatics, Federal University of Rio Grande do Sul, Porto Alegre 91501-970, Brazil

**Keywords:** long non-coding RNA, *Candida albicans*, infection, transcriptomics, guilt by association

## Abstract

*Candida albicans* is one of the most commonly found species in fungal infections. Due to its clinical importance, molecular aspects of the host immune defense against the fungus are of interest to biomedical sciences. Long non-coding RNAs (lncRNAs) have been investigated in different pathologies and gained widespread attention regarding their role as gene regulators. However, the biological processes in which most lncRNAs perform their function are still unclear. This study investigates the association between lncRNAs with host response to *C. albicans* using a public RNA-Seq dataset from lung samples of female C57BL/6J wild-type *Mus musculus* with induced *C. albicans* infection. The animals were exposed to the fungus for 24 h before sample collection. We selected lncRNAs and protein-coding genes related to the host immune response by combining the results from different computational approaches used for gene selection: differential expression gene analysis, co-expression genes network analysis, and machine learning-based gene selection. Using a guilt by association strategy, we inferred connections between 41 lncRNAs and 25 biological processes. Our results indicated that nine up-regulated lncRNAs were associated with biological processes derived from the response to wounding: 1200007C13Rik, 4833418N02Rik, Gm12840, Gm15832, Gm20186, Gm38037, Gm45774, Gm4610, Mir22hg, and Mirt1. Additionally, 29 lncRNAs were related to genes involved in immune response, while 22 lncRNAs were associated with processes related to reactive species production. These results support the participation of lncRNAs during *C. albicans* infection, and may contribute to new studies investigating lncRNA functions in the immune response.

## 1. Introduction

Species of *Candida* are widely found in the human microbiota. Although mostly harmless for healthy individuals, these fungi can cause severe infection in immunocompromised individuals [1]. Candidemia (bloodstream candidiasis) and invasive candidiasis are life-threatening and recurrent in hospital environments, affecting patients undergoing immunosuppressive therapy, hemodialysis, recovering from organ transplantation, and even debilitated low-birth-weight infants [2,3]. Although different *Candida* species are clinically relevant, such as *Candida glabrata*, *C. parapsilosis*, *C. tropicalis* [4], and *C. auris* [5], *C. albicans* remains the most common pathogen responsible for candidemia [6].

Long non-coding RNA (lncRNA) are molecules with a length longer than 200 nucleotides, subdivided according to their genomic localization or transcription process: antisense, bidirectional, intergenic, intronic, or sense-overlapping lncRNA [7]. These transcripts are essential to several cellular processes related to gene regulation, such as enhancement or interference during transcription, as well as post-transcriptional modifications [8]. A single lncRNA can also target different genes and molecules and therefore participate in more than one form of regulation.

The extent of lncRNA regulatory contribution to host defense against pathogens, or eventually aggravating the infection, remains a research topic [9]. Some reports indicate specific lncRNAs as potential transcription regulators during infections promoted by pathogens such as *Mycobacterium tuberculosis* [10] and SARS-CoV-2 [11]. Only a few studies have examined lncRNA’s association with fungal infections. Human lncRNAs RP11-588G21.2 and RP11-394I13.1 have been identified and proposed as potential biomarkers for *C. albicans* infection [12]. Hou et al. [13] recently reported the lncRNA lnc-CCL3L3-1:1 as potentially related to the human inflammatory response to β-glucan, while analyzing gene expression of CD14+ monocytes stimulated by *C. albicans* cell wall polysaccharide.

According to Ensembl BioMart data [14], 42% of the lncRNAs in *Homo sapiens* (GRCh38.p13) are annotated as novel transcripts, indicating these molecules are yet to be characterized. At the same time, the number reaches 64% for the model organism *Mus musculus* (GRCm39) when also including novel transcripts from predicted genes. Even among annotated lncRNA, less than 3% have known functions [15].

To understand how lncRNAs can participate in host immune response during *C. albicans* infection, we propose new associations between these molecules and biological processes in *M. musculus* by using a public RNA-seq dataset and multiple bioinformatic approaches. Our results suggest the association of 41 lncRNAs not previously described in *C. albicans* infection. The present study shows that these lncRNAs are associated with multiple biological processes related to inflammation, response to wounding, and host defense.

## 2. Materials and Methods

**Figure 1:**
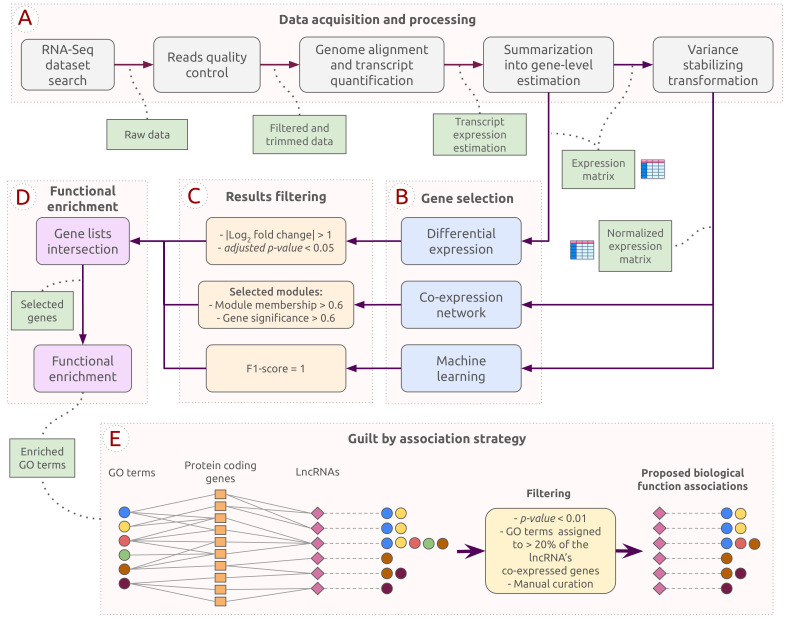
Pipeline representing the analyses performed in this study, including the following steps: (**A**) Data acquisition and processing; (**B**) Gene selection; (**C**) Results filtering; (**D**) Intersection and functional enrichment analysis; and (**E**) Guilt by association strategy.

The pipeline employed in this work was designed to explore potential functional roles of lncRNAs during *C. albicans* infection. Figure 1 provides the steps for all analyses performed and the chosen thresholds. The pipeline was divided into stages consisting of data acquisition and processing, the three methods for gene selection, results filtering and intersection, functional enrichment analysis, and a guilt by association strategy. This section will subsequently detail the tools and parameters determined for each step. The source code and hyperparameters used in the experiments and results can be accessed on GitHub: https://github.com/sbcblab/candida-infection-pipeline, (accessed on 3 May 2021).

### 2.1. Samples and Data Preprocessing

The dataset used in this study is available at NCBI Gene Expression Omnibus (GEO) under the identifier GSE119853 [16]. It consists of 19 lung samples from female C57BL/6J wild-type *M. musculus*—11 samples from control animals and 8 samples from animals exposed to *C. albicans* for 24 h previous to sample collection.

For preprocessing the raw data, tools FastQC (v. 0.11.09, https://www.bioinformatics.babraham.ac.uk/projects/fastqc, accessed on 3 May 2021) and Trimmomatic (v. 0.39) [17] were applied to perform quality control and trimming or removal of low-quality sequence reads. Adapter sequences indicated by FastQC were also removed.

### 2.2. Gene Expression Matrix and Analysis

After preprocessing, raw reads were mapped against the *M. musculus* reference genome (GRCm39) with the STAR (v. 2.7.9A) [18] aligner through RSEM (v. 1.17) [19]. RSEM was used to quantify the transcripts previously found. By using the tximport package (v. 1.24.0) [20] implemented in R, RSEM results were summarized in a gene-level matrix. The summarized matrix (*m* genes x *n* samples) was imported to the DESeq2 package (v. 1.36.0) [21] for gene expression analysis. Genes with $|\;\log_{2}fold\;change\;|\;>1$ and adjusted *p*-value < 0.05 were considered differentially expressed genes (DEGs). The *Benjamini–Hochberg* method was used to adjust the *p*-value.

A normalized version of the gene expression matrix was obtained using the *varianceStabilizingTransformation* function (VST) from the DESeq2 package. The resulting normalized matrix was further used as input to construct the co-expression networks and perform the machine learning analysis.

### 2.3. Co-Expression Network Construction and Analysis

The WGCNA package (v. 1.71) [22] was employed to construct and analyze a weighted gene co-expression network. The normalized matrix was utilized as input, and the *goodSamplesGenes* function was applied to remove genes with low variance or those infrequent amongst all samples. The following settings were chosen for the generation of the network: signed network type; β=20 (soft-threshold parameter chosen based on the scale-free topology criterion, $r^{2}$ > 0.8); deepSplit=2; mergecutHeight=0.3; minmodulesize=50.

A Pearson correlation between trait information and module eigengene (ME) was performed to investigate whether the network modules were significantly associated with the clinical condition. ME is considered the first principal component of the module and summarizes the module’s overall expression. For further analysis, we selected the module with the strongest correlation coefficient related to the disease and control condition.

To identify the most significant genes related to the clinical conditions, we considered the measures of module membership (MM), which represents the correlation between gene expression and ME, and the gene significance (GS), which describes the correlation between gene expression and the external trait. The mentioned measures were used to filter the most relevant genes for each module using cut-offs of MM>0.6 and GS>0.6.

### 2.4. Support Vector Classification

A machine learning approach was employed to select the genes most likely related to each condition. With the normalized matrix serving as input, each gene was individually evaluated by a support vector classification (SVC) algorithm available at SciKit Learn (v. 1.1.2, https://scikit-learn.org, accessed on 30 July 2021) [23]. Due to the reduced sample size, the leave-one-out method was used as a cross-validation strategy to divide the normalized gene expression values into train and test subsets. Using the f1-score to evaluate classification success, we selected those genes whose prediction reached f1-score = 1.0. This rigorous threshold was established to reinforce the selection of only genes for which the expression value range did not overlap between healthy and infected samples, i.e., genes distinctly expressed under all the samples from both conditions.

### 2.5. Functional Enrichment Analysis

The selected genes from both co-expression modules were submitted to functional enrichment analysis using the topGO package (v. 2.48.0) [24] to investigate the biological processes associated with the data. Only Gene Ontology (GO) terms with an adjusted *p*-value < 0.05 were retrieved. The method used to correct the *p*-value was the false discovery rate (FDR). The topGO results were manually investigated for further discussion.

Moreover, we explore the functional enrichment results associated with the lncRNAs. To perform that, we created a subnetwork considering only the lncRNAs and their protein-coding genes’ first-degree neighbors. These lncRNA–coding gene interactions must show a moderate or strong correlation coefficient (≥0.4). After, functional enrichment was performed on the subnetwork. The GO terms assigned to the co-expressed protein-coding genes were summarized for each lncRNA considering only the resulting GO terms with adjusted *p*-value < 0.01 and enriched for at least 20% of protein-coding genes interacting with each lncRNA. These results were manually inspected and selected for the guilt by association strategy.

### 2.6. Gene Biotype Classification

The biomaRt package (v. 2.52.0) [25] provided biological classification information about the different transcripts identified. Afterward, we performed analyses with CPC (v. 2.0 beta) [26] and RNAsamba (web version, https://rnasamba.lge.ibi.unicamp.br, accessed on 6 June 2022) [27] to validate the genes classified as lncRNAs. We used the genes’ complementary DNA (cDNA) sequences as input in both tools. Predictions for each sequence were summarized to establish a consensus for each gene, considering every isoform and both tools. Manual curation was performed using NCBI Gene database (https://www.ncbi.nlm.nih.gov/gene, accessed on 9 June 2022) considering “*Mus musculus*” and “Gene type” information for those with “RefSeq Status = Validated”. lncRNAs with missing information were disregarded.

## 3. Results

The selected dataset was processed and analyzed to identify lncRNAs expressed during *C. albicans* infection. After the data acquisition and the processing steps described in Section 2.1 and Section 2.2 (see also Figure 1A), we obtained an expression matrix of 27,677 genes. The expression matrix was used as input for the three gene selection approaches (differential expression analysis, co-expression network analysis, and machine learning-based gene selection; Figure 1B, C). The resulting genes from each approach were intersected for further investigations (Figure 1D). A potential biological function was attributed to each lncRNA by considering their co-expressed first-degree neighbors using a guilt by association strategy. In the end, 64 lncRNAs were selected and investigated to infer which biological processes they could be associated with (Figure 1E).

### 3.1. Co-Expression Networks

**Figure 2:**
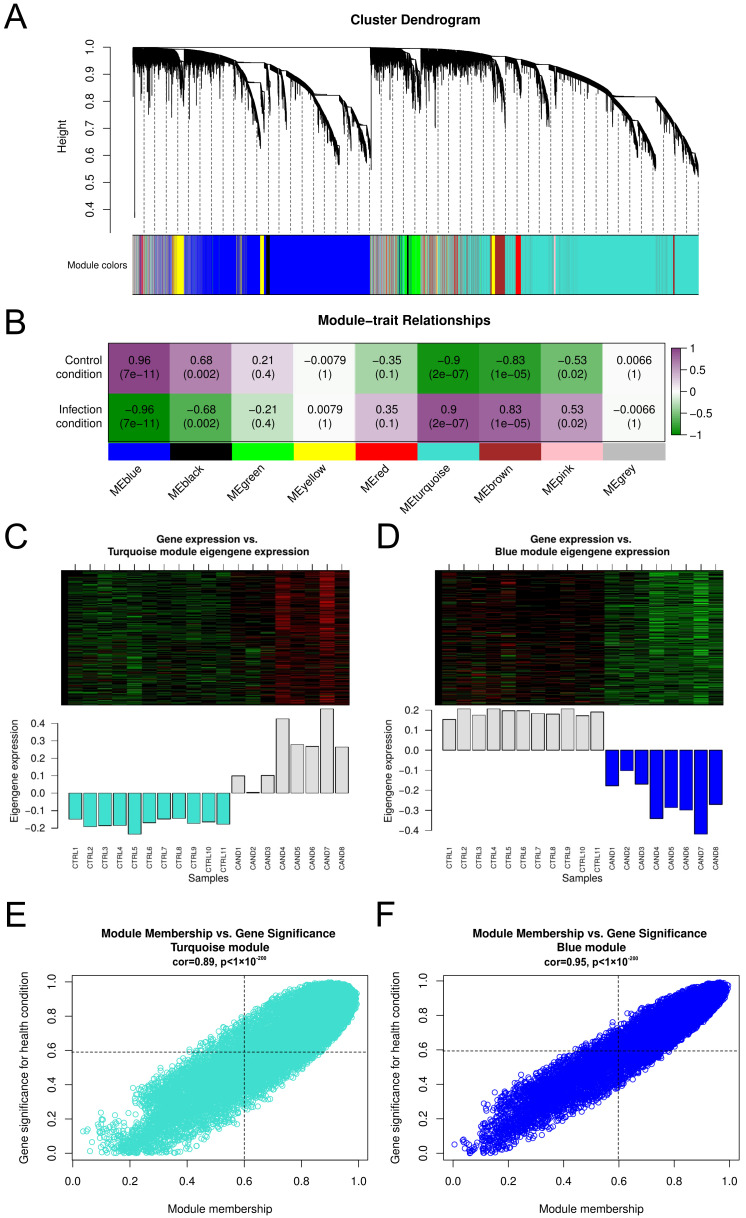
(**A**) Cluster dendrogram representation of the co-expression network modules. (**B**) Correlation between the eigengene modules and the health condition trait. (**C**) Heatmap presenting the sample gene expression for the turquoise module. The barplot shows the module eigengene expression value associated with each sample. (**D**) Heatmap presenting the sample gene expression for the blue module. The barplot shows the module eigengene expression value associated with each sample. (**E**) Correlation between gene significance (GS) and MM for genes in the turquoise module. (**F**) Correlation between gene significance (GS) and MM for genes in the blue module.

We constructed a co-expression network to uncover the main biological pathways related to the host response during *C. albicans* infection (Figure 1B). Highly interconnected genes were clustered into eight modules represented by different colors (Figure 2A). The co-expression network approach allows the inference of gene-gene relationships by identifying modules strongly related to the health condition (infection or control). The results showed that multiple modules are related to each condition, whereas turquoise (r=0.9, *p*-value = $2\times 10^{-07}$) and blue (r=0.96, *p*-value = $7\times 10^{-11}$) modules presented the highest correlation coefficient values associated with infection and control, respectively. To further explore these results and confirm the significance of turquoise and blue modules, eigengene expression across individual samples was evaluated, while heatmaps show the gene expression of the selected modules (Figure 1D and Figure 2C). Based on these results, we consider the blue and turquoise modules for further analysis.

In these modules, MM and GS measures allowed the identification of genes strongly related to the infection and control conditions (GS and MM > 0.6) (Figure 2E,F), resulting in a total of 10,938 genes selected from the turquoise module (1876 lncRNAs) and 8896 genes from the blue module (1207 lncRNAs).

### 3.2. Differentially Expressed Genes

**Figure 3:**
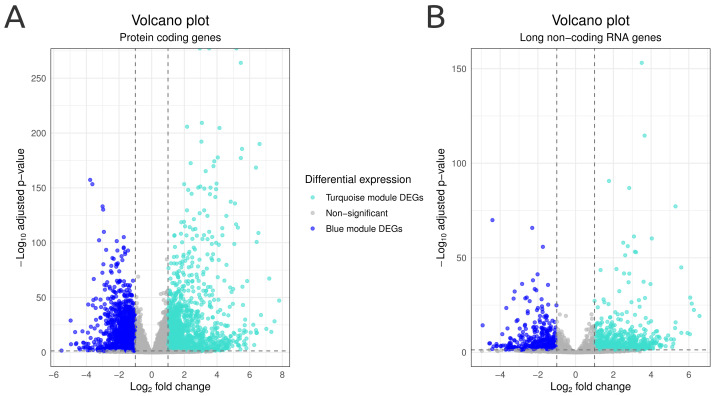
Volcano plots showing DEGs results, with adjusted *p*-value in the y-axis and $|\log_{2}fold\;change|$ in the x-axis. Genes are divided according to their biotype: (**A**) protein-coding or (**B**) long non-coding RNA. Blue and turquoise colors represent DEGs assigned to blue (control condition) or turquoise (infection condition) modules.

The gene expression profiles of infection samples were compared to control samples, resulting in 5576 DEGs (3509 up- and 2463 down-regulated) identified between the conditions (Figure 1C). Protein-coding genes were the most abundant biotype identified in the DEGs (57.4%), with 1710 genes up-regulated and 1719 down-regulated in *Candida*-infected samples, as exhibited by the volcano plot in Figure 3A. The lncRNA group represents 20.8% of all DEGs, where 800 were up-regulated and 441 down-regulated in the infected animals (Figure 3B). In the volcano plot figures, all DEGs are colored according to their assigned co-expression module. Up-regulated protein-coding and lncRNA DEGs were only found in the turquoise module, while all down-regulated protein-coding and lncRNA DEGs belong to the blue module. The remaining biotypes identified correspond to pseudogenes, genes to be experimentally confirmed, immunoglobulin variable chains, and T-cell receptors.

### 3.3. SCV-Based Gene Selection

Selecting genes with a consistent expression value range among all samples was essential to increase the chances of including only genes associated with each health condition. Every gene was individually classified with the sci-kit learn SVC algorithm according to its VST normalized expression matrix (Figure 1B,C). For the turquoise module, the prediction of sample condition reached f1-score = 1 for 1438 genes, meaning that those genes could perfectly separate infected and control samples during the tests. As for the blue module, 1095 genes classified the health condition with maximum accuracy.

### 3.4. Candidate Genes Prioritization

**Figure 4:**
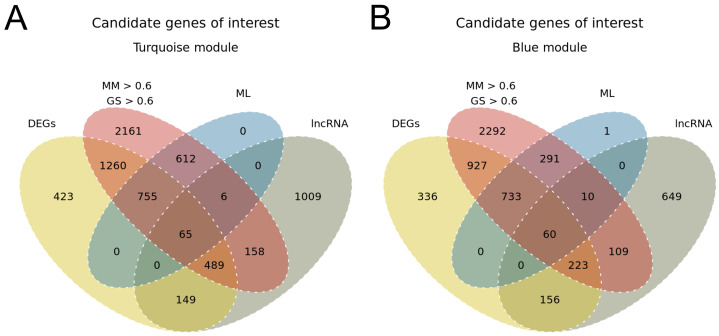
Venn diagram illustrating the number of genes selected by different approaches: DEGs, MM and GS>0.6, ML, and filtering for lncRNA biotype. (**A**) All genes from the turquoise module. (**B**) All genes from the blue module.

To narrow down the candidate genes of interest, we cross-referenced the results from all approaches previously applied: DEGs analysis, module identification followed by gene selection using MM and GS>0.6 as the cut-off, and machine learning-based gene selection (Figure 1C,D). The intersected gene lists resulted in 820 genes from the turquoise module, including 65 lncRNAs. Figure 4A presents the number of selected genes through each approach and its intersections considering the turquoise module. The same intersection was performed for the blue module, where 793 genes were selected, including 60 lncRNAs (Figure 4B).

Afterward, the 125 selected genes classified as lncRNAs were validated to confirm their assigned gene type, resulting in the exclusion of 12 lncRNAs. Fifty-eight lncRNA from the turquoise module and fifty-five from the blue module remained.

The confirmed lncRNAs were used as seeds to filter the blue and turquoise modules and create different subnetworks. These subnetworks included only the lncRNAs’ first-degree neighbors, showing a moderate or strong correlation coefficient (r≥0.4) [28]. When the subnetworks were created, not all lncRNAs showed first-degree interactions following the cut-off criterion. Consequentially, 51 lncRNAs from the turquoise network remained, forming a subnetwork with 657 nodes and 4479 edges. The blue subnetwork included 111 nodes (14 representing lncRNAs) and 129 edges. Only the genes included in those networks were considered for the following analyses.

### 3.5. Functional Enrichment Analysis

**Figure 5:**
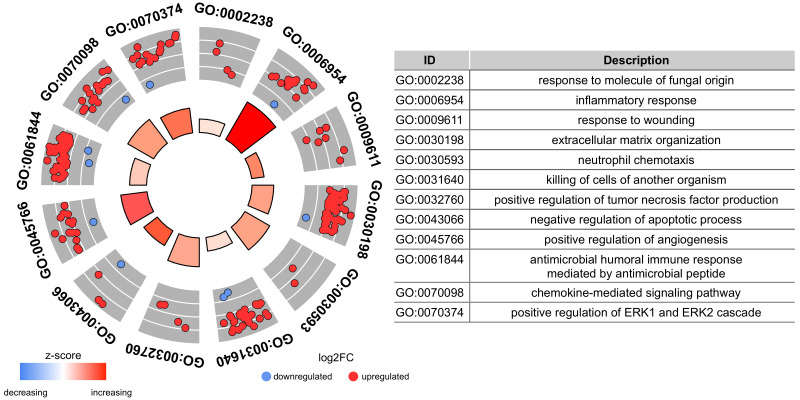
The table on the right side shows the top 15 GO terms enriched for the selected genes from both modules analyzed. On the left, the outer circle’s scatter plots represent the GO terms identified and the regulation of its associated genes, based on the $\log_{2}\;fold\;change$ values. The z-scores showed in the inner circle were calculated for each BP as *z*-score=(up-regulatedgenes−down-$regulated\;genes)/\sqrt{total\;genes}$.

Functional enrichment analysis was performed to investigate the biological processes related to the previously selected genes from both networks and how they might be associated with *C. albicans* infection. We highlighted enriched GO terms, such as response to molecules of fungal origin, inflammatory response, and response to wounding, among others. The GO terms are represented in Figure 5, which includes information on the regulation associated with each gene set. Genes not presented in any GO term were not used to infer lncRNA-associated roles.

**Figure 6:**
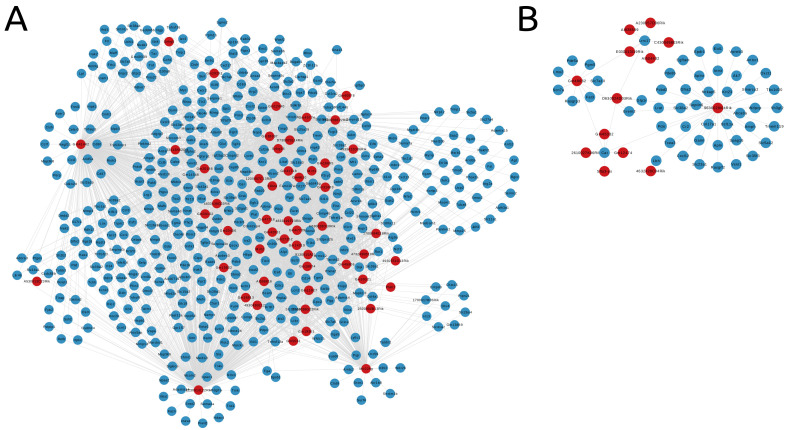
Subnetworks representing the selected protein-coding genes (in blue) and lncRNAs (in red). (**A**) Turquoise module. (**B**) Blue module.

In total, 471 protein-coding genes from the turquoise module were identified in the functional enrichment. Those genes formed the final subnetwork alongside the remaining 51 lncRNAs and 3568 edges, as shown in Figure 6A. For the blue module, one lncRNA lost all its connections and was excluded, leaving the subnetwork with 13 lncRNA genes, in addition to the 50 protein-coding genes and 66 edges (Figure 6B).

## 4. Discussion

Unraveling the molecular mechanisms related to lncRNAs has been challenging, primarily due to the heterogeneous nature of this molecule and its relatively low levels of gene expression [29]. Furthermore, lncRNA sequences are less conserved across species than protein-coding genes [29], and structural domains that determine their functionality must be better understood.

Here, we take several steps to ensure data quality and combine stringent filtering criteria using various integrative approaches to achieve our results. None of the 64 selected lncRNAs (51 up- and 13 down-regulated) have been previously associated with *C. albicans* infection. However, 18 lncRNAs were mentioned in studies related to different health conditions (Table 1), including infections, lung diseases, and injury.

**Figure 7:**
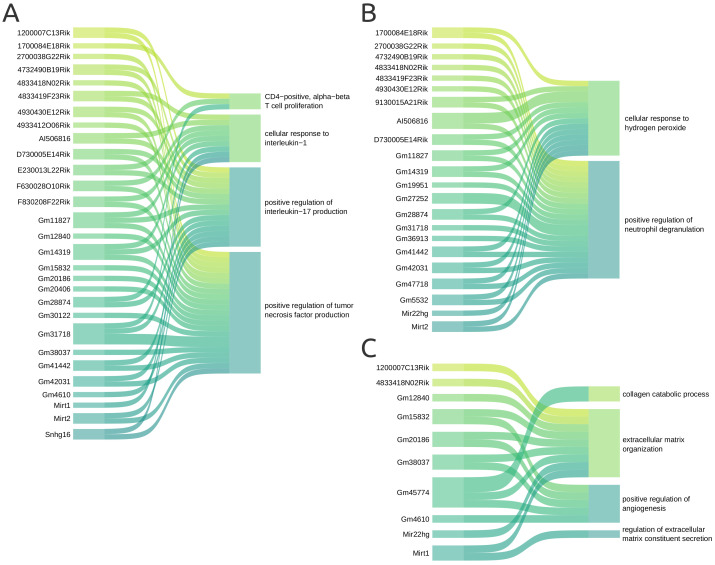
Sankey plot representing the GO terms inferred to the selected lncRNAs. The GO terms were grouped according to its biological function. (**A**) GO terms associated with the inflammatory response. (**B**) GO terms associated with reactive oxygen species.(**C**) GO terms involved in response to wounding.

For the guilt by association approach, we selected representative GO terms corresponding to the protein-coding genes interacting with each lncRNA in the co-expression network. This approach assumes that genes that share some interaction or expression pattern are more likely to participate in similar processes or even the same functions [30], and has been previously used to explore lncRNAs without annotated functions [31]. It also enables the inference of unknown biological processes for genes when their co-expressed pairs already have described functions. The most relevant associations are shown in Figure 7 and will be discussed below. Supplementary Figure S1 contains all the proposed associations between the 41 lncRNAs and 25 GO terms.

### 4.1. Inflammatory and Immune Response Might Be Promoted by lncRNA

According to the results, F630028O10Rik interacted with protein-coding genes enriched in inflammatory cytokine-derived GO terms, such as positive regulation of tumor necrosis factor (TNF) production and cellular response to interleukin-1 (IL-1). Both TNF and IL-1 are pro-inflammatory cytokines produced and secreted by cells such as macrophages and mediated through the NF-κB pathway. As presented in Figure 7A, we propose that some lncRNAs may be involved in both biological processes, namely Snhg16, F830208F22Rik, Gm14319, Gm11827, and Gm31718.

Furthermore, 17 lncRNAs were connected to positive regulation of interleukin-17 (IL-17) production (Figure 7A). IL-17 is a pro-inflammatory cytokine secreted by T helper 17 cells, which derive from the T helper (also known as CD4-positive). The GO terms CD4-positive and alpha-beta T cell proliferation were also enriched during the functional analysis, and the lncRNAs 1700084E18Rik, E230013L22Rik, and Gm28874 are connected to it through the protein-coding genes they interact with. This indicates those lncRNAs may participate in both proliferation and differentiation of CD4-positive cells. In addition, lncRNAs cell-specific expression patterns have been observed during proliferation in human T helper cell cultures [32].

The lncRNA Mirt2 was associated with positive regulation of IL-17 and TNF production, as shown in Figure 7A. In this context, Mirt2 regulates excessive inflammatory response in mice macrophage after lipopolysaccharide stimulus [33] (Table 1). Meanwhile, the lncRNA E230013L22Rik was linked to the GO term inflammatory response to antigenic stimulus. E230013L22Rik was previously reported as a potential regulator in the development of sepsis in mice [34]. Zou et al. inferred the lncRNA association with pro- and anti-inflammatory cytokines, besides inflammasome activation (Table 1).

GO terms related to immune response activation were also enriched in our analysis. Candidalysin can activate immune response by inducing the MAPK signaling pathway via MEK1/2 and ERK1/2 in endothelial cells [35]. Likewise, it can activate the immune system by inducing chemokines that mediate neutrophil and monocyte chemotaxis to the wound site [35,36]. At last, *C. albicans* cell wall components such as β-1,3-glucans, β-1,6-glucans, and mannoproteins can be identified by pattern recognition receptors (PRR) and trigger the immune response [37].

### 4.2. lncRNAs Related to Reactive Species Production during Host Defense

Neutrophils and macrophages are the first recruited cells to neutralize *C. albicans* by producing cytotoxic molecules, such as reactive oxygen species (ROS) and nitric oxide (NO) [38]. Reactive species generation is effective in fighting against microbial infections, being produced when the pathogen is recognized and during the response process [39]. Macrophages can create a toxic environment for the engulfed pathogen with an excessive amount of copper ion, generating ROS [40]. The 2500002B13Rik lncRNA, now denominated iNOS Transcriptional Regulatory Intergenic LncRNA Locus (Nostrill), was recently characterized [41]. Mathy et al. discovered that Nostrill overexpression was associated with up-regulation of the inducible nitric oxide synthase (iNOS) gene and NO production upon challenging microglia cells with bacterial LPS (Table 1).

Similarly, neutrophils also utilize reactive species by using both O_2_-dependent and O_2_-independent mechanisms to attack invading microorganisms [39]. Unlike macrophages, they take action by externalizing granules containing cytotoxic substances, performing a process known as degranulation [42]. Positive regulation of neutrophil degranulation was the second most frequent GO term associated with the lncRNA from our network (Figure 7B). Neutrophils have a critical role in host defense against fungal infections, killing the pathogen by different strategies, such as phagocytosis, oxidative bursts, and forming extracellular traps. All the mentioned strategies rely on reactive species [43,44]. Phagocytosis stimulates the generation of ROS such as hydrogen peroxide, observed in Figure 7B. Several lncRNAs also interact with genes enriched in the cellular response to the hydrogen peroxide ($H_{2}$$O_{2}$) process (Figure 7B). $H_{2}$$O_{2}$ is essential to immune response, as it promotes neutrophil recruitment and chemotaxis to the wounding site [45]. At low levels, $H_{2}$$O_{2}$ supports normal physiological processes by inducing signaling pathways [46]. However, under certain stimuli, such as growth factors or chemokines, $H_{2}$$O_{2}$ production is increased, leading to reversible oxidation of specific proteins, which alters their activity, localization, and interactions [46]. Consequently, these modified proteins contribute to orchestrating different processes, such as cell proliferation, differentiation, migration, and angiogenesis [46]. However, prolonged exposure to high ROS levels causes non-specific oxidation, impairing macromolecule function and leading to tissue damage [46].

### 4.3. Response to Wounding Healing Is Potentially Mediated by lncRNA

The functional enrichment analysis revealed critical biological processes associated with the host’s response to infection. From the sixty-four lncRNAs, only two were directly found in the GO enrichment gene sets, both up-regulated and assigned to the “response to wounding” GO term: Mir22hg (ENSMUSG00000085148) and AI506816 (ENSMUSG00000105987). Response to wounding is expected, given that infection by *C. albicans* requires invasion of epithelial and endothelial tissues [47]. After adhering to the epithelial cells, *C. albicans* hyphae secrete candidalysin, a toxin known to damage epithelial cells and cause cellular stress to initiate the infection [48,49]. Eventually, tissue damage can be aggravated due to an exaggerated inflammatory response to control the infection. Additionally, during pathogen infection, rapid generation of reactive species is crucial to the host defense, but prolonged exposure is detrimental to the surrounding tissue [49]. The wound healing process includes angiogenesis, extracellular matrix (ECM) organization, and inflammation processes also enriched among the lncRNAs.

The Sankey plot in Figure 7C represents the associations between GO terms involved in processes related to wounding response and the lncRNAs. Nine lncRNAs interact with protein-coding genes involved in ECM organization: 1200007C13Rik, 4833418N02Rik, Gm12840, Gm15832, Gm20186, Gm38037, Gm45774, Mir22hg, and Mirt1. Four of the mentioned lncRNAs also interact with genes associated with positive regulation of angiogenesis, namely Gm15832, Gm4610, Gm38037, and Gm45774, with the addition of Gm4610. Meanwhile, Gm45774 interacts with genes involved in the collagen catabolic process and Gm4610 with a gene linked to regulating extracellular matrix constituent secretion. Mir22hg was already directly associated with response to wounding within the functional enrichment results, which supports the result obtained with the designed strategy.

The angiogenic process is essential to effective wound healing as it promotes vascularization for the new tissue being assembled. A study associated angiogenesis with *C. albicans* and candidalysin secretion due to increased levels of fibroblast growth factor (FGF-2) in infected animals [50]. Moreover, the authors observed a greater mortality rate in animals treated with FGF-2 and hypothesized that this process might improve the fungus pathogenicity. Yet, fibroblasts are crucial to wounding response, as these molecules synthesize a new ECM [36]. In addition, the negative regulation of the apoptotic process was enriched in our analysis. While this biological process can be linked to the endothelial cell proliferation that occurs during wound healing, as previously reported [51,52], it has been observed that apoptosis inhibition could also be a host solution to keep more viable macrophages, increasing its defense [52].

F630028O10Rik was associated with reduced angiogenesis in lung cancer [53]. This lncRNA was also associated with increased spine chord injury severity due to the inflammatory response [54]. Xu et al. observed that the gene acted in TLR4-induced pyroptosis and served as a competing endogenous RNA; its overexpression favored the up-regulation of Col1a1 (collagen, type I, alpha 1), a gene involved in inflammation. Interestingly, up-regulation of F630028O10Rik was also observed in our analysis, while the TLR4 gene was not considered a DEG due to its expression values ($\log_{2}fold\;change=0.77$). As a result of our functional enrichment analysis, no pyroptosis-derived GO term was assigned. Additionally, Col1a1 was slightly down-regulated ($\log_{2}fold\;change=-0.58$, adjusted *p*-value = $5.77\times 10^{-07}$) in the *C. albicans* infected mice. Although both datasets describe different health conditions, the divergent transcript patterns mentioned indicate that F630028O10Rik may also regulate other genes, as lncRNAs can interact with different targets [55].

**Table 1:** Information regarding the lncRNA reported in the literature, as well as the module, gene significance (GS), module membership (MM), and $\log_{2}fold\;change$ ($\log_{2}FC$) from all selected lncRNA. Myocardial infarction: MI; lipopolysaccharide, LPS.

Gene Symbol	Literature			Expression Data			
	Biological Process	Condition	Organism	Module	GS	MM	$\log_{2}\mathrm{FC}$
1200007C13Rik				Turquoise	−0.97	0.93	5.59
1700084E18Rik				Turquoise	−0.92	0.80	1.87
2500002B13Rik	Increases expression of the inducible nitric oxide synthase (iNOS) gene and nitric oxide production [41]	LPS stimulus	*M. musculus*	Turquoise	−0.91	0.72	1.84
2700038G22Rik				Turquoise	−0.88	0.81	1.27
4732490B19Rik				Turquoise	−0.94	0.87	4.00
4833418N02Rik				Turquoise	−0.93	0.90	1.52
4833419F23Rik				Turquoise	−0.93	0.81	2.31
4833438C02Rik				Turquoise	−0.92	0.83	1.17
4930430E12Rik				Turquoise	−0.91	0.84	3.43
4930551O13Rik				Turquoise	−0.85	0.81	2.40
4933412O06Rik				Turquoise	−0.95	0.92	4.09
9130015A21Rik				Turquoise	−0.96	0.81	3.40
A530013C23Rik	Associated with gut microbiota-dysbiosis [56]	Gut microbiota transplant	*M. musculus*, *H. sapiens*	Turquoise	−0.84	0.92	1.89
AI506816	Up-regulated during mice second pregnancy in comparison to first pregnancy [57]	Multiparity	*M. musculus*	Turquoise	−0.96	0.85	1.32
D730005E14Rik				Turquoise	−0.97	0.87	2.84
E230013L22Rik	Up-regulated during sepsis [34]	Septic acute lung injury		Turquoise	−0.96	0.97	3.09
F630028O10Rik	Promotes angiogenesis inhibition [53]	Lung cancer	*M. musculus*	Turquoise	−0.95	0.92	2.16
	Enhanced microglial pyroptosis by activating the PI3K/AKT pathway	Spinal cord injury	*M. musculus*, *H. sapiens*				
F830208F22Rik				Turquoise	−0.90	0.94	3.14
Gm11827				Turquoise	−0.97	0.83	3.20
Gm12840				Turquoise	−0.95	0.92	2.56
Gm14319				Turquoise	−0.91	0.81	2.14
Gm15832	Up-regulated in infarct border zone samples [58]	MI	*M. musculus*	Turquoise	−0.96	0.93	2.76
Gm16685 (NAIL)	Proinflammatory response regulation [59]	Ulcerative colitis	*M. musculus*	Turquoise	−0.92	0.97	3.96
Gm19951				Turquoise	−0.93	0.77	1.74
Gm20186				Turquoise	−0.94	0.97	2.84
Gm20406				Turquoise	−0.95	0.93	5.30
Gm27252				Turquoise	−0.97	0.86	2.62
Gm28874				Turquoise	−0.93	0.81	1.82
Gm29292				Turquoise	−0.81	0.88	2.42
Gm30122				Turquoise	−0.95	0.89	1.48
Gm31683				Turquoise	−0.88	0.91	2.40
Gm31718				Turquoise	−0.96	0.85	3.14
Gm36913				Turquoise	−0.94	0.80	4.77
Gm38037				Turquoise	−0.91	0.91	1.93
Gm39321				Turquoise	−0.90	0.83	2.26
Gm40578				Turquoise	−0.87	0.93	3.71
Gm41442	Potential regulator during sepsis [34]	Sepsis	*M. musculus*	Turquoise	−0.95	0.97	4.03
Gm42031				Turquoise	−0.98	0.88	3.65
Gm45606				Turquoise	−0.90	0.85	1.12
Gm45774				Turquoise	−0.96	0.96	5.29
Gm4610				Turquoise	−0.96	0.88	2.80
Gm47709				Turquoise	−0.90	0.94	4.82
Gm47718				Turquoise	−0.95	0.78	3.28
Gm5532				Turquoise	−0.89	0.76	1.87
Il1bos				Turquoise	−0.93	0.95	2.95
Mir22hg	Response to cycloheximide and cisplatin stress [60,61]	Chemical stress	*H. sapiens*	Turquoise	−0.98	0.93	1.76
Mirt1	Association with genes involved in left ventricular remodeling [62]	MI	*M. musculus*	Turquoise	−0.96	0.89	2.52
	NF-κB activation [63]	MI	*M. musculus*				
Mirt2	Association with genes involved in left ventricular remodeling [62]	MI	*M. musculus*	Turquoise	−0.96	0.89	3.60
	Reduced inflammatory response [33]	LPS stimulus	*M. musculus*				
	Reduced apoptosis [64]	Accute MI	*M. musculus*				
Platr7				Turquoise	−0.87	0.86	3.03
Snhg16	Enhanced the LPS-induced inflammatory pathway [65]	LPS stimulus	*M. musculus*	Turquoise	−0.89	0.96	1.03
Snhg5	Decreased target mRNA degradation by STAU1 [66]	Colorectal cancer	*M. musculus*	Turquoise	−0.88	0.93	1.12
2610027K06Rik				Blue	0.96	0.97	−2.01
4632428C04Rik				Blue	0.95	0.96	−2.08
9630028I04Rik				Blue	0.96	0.98	−3.23
A230057D06Rik				Blue	0.95	0.90	−2.43
AI504432				Blue	0.97	0.94	−1.73
AI838599	Prediction: regulation of the TNFα-NF-κB signaling pathway [67]	Diabetic neuropathy	*M. musculus*	Blue	0.97	0.93	−2.30
C430049B03Rik	Demarking between embryonic and extra-embryonic mesoderm [68]	Cardiogenesis	*M. musculus*	Blue	0.96	0.92	−2.26
D630024D03Rik				Blue	0.96	0.94	−2.83
E030013I19Rik				Blue	0.93	0.88	−2.05
Gm12474	Unknown. Possibly has a role in regulating the *gsdmd* gene [69]	Diabetic periodontitis	*M. musculus*	Blue	0.94	0.94	−2.59
Gm45332				Blue	0.94	0.96	−1.78
Gm48662				Blue	0.98	0.96	−4.40
Sfta3-ps				Blue	0.95	0.99	−1.46

## 5. Conclusions and Perspectives

The understanding of *C. albicans*–host interactions is crucial to investigate new forms of diagnostic and therapeutic methodologies. Molecules such as lncRNAs are still little studied in this context, which results in limited reports asserting their importance during the immune response to fungal infections. A recent review discussed how lncRNAs reportedly relevant for host defense against other pathogens could also participate in the immune response against *C. albicans*, as well as be used in host-derived therapies [70]. The authors proposed that lncRNAs could be interesting targets for therapy focusing on different defense mechanisms, such as tissue barrier and inflammation. Mirt2 was proposed as a lncRNA potentially related to the induction of cytokines, an essential process during the immune response to *C. albicans*.

In this study, we infer biological functions in which lncRNAs may participate during host immune defense against *C. albicans* infection. For that, we used a guilt by association strategy. Many of the selected lncRNAs are not described in the literature, and none have been reported in fungal infection.

Our presented results suggest that 46 lncRNAs might participate in processes related to 25 selected GO terms (Supplementary Figure S1) and should be further investigated. Among the nine lncRNAs associated with wounding healing processes, Gm45774 was linked to three of the four GO terms selected, as shown in Figure 7C. Three out of the twenty-nine associated lncRNAs could be highlighted in the inflammation processes: Gm31718, Gm11827, and Gm14319. While Gm31718 was linked to all the selected GO terms, Gm11827 and Gm14319 were associated with the GO terms regarding cytokines: cellular response to interleukin-1, positive regulation of interleukin-17 production, and positive regulation of tumor necrosis factor production (Figure 7A). Some GO terms were associated with several lncRNAs, highlighting positive regulation of tumor necrosis factor production, positive regulation of neutrophil degranulation, and immunoglobulin-mediated immune response (Supplementary Figure S1). These findings might provide insight into the role of lncRNA during *C. albicans* infection. While the selected lncRNAs are up-regulated during the host’s defense against *C. albicans*, we can not determine whether they are expressed due to the presence of the fungi or as part of the general immune response. More data analysis, including different infected organs samples and periods throughout the infection, would be necessary to evaluate lncRNA specificity.

Mortality related to *C. albicans* infection is a challenge worldwide, with significant incidence and recurrence rates in vulnerable patients. Growing evidence suggests that lncRNAs play a critical role in the immune response during infections. Exploring these molecules further could give us strategic options for therapeutic treatments for fungal infections. The lncRNAs uncovered in this work can be used as a starting point to pave the way for future research to illustrate the role of these molecules in defense mechanisms. Furthermore, designing a more extensive investigation to help unravel the different modes of action of lncRNAs and understanding the miRNA–lncRNA–mRNA axis may help to solve the role of lncRNAs in the immune response and as promising molecules for future targeted therapy.

## Data Availability

The source code used in the experiments can be accessed on GitHub: https://github.com/sbcblab/candida-infection-pipeline, accessed on 3 May 2021.

## Supplementary Materials

The following supporting information can be downloaded at: https://www.mdpi.com/article/10.3390/genes14020251/s1, Figure S1: Sankey plot representing each selected lncRNA (right side) and the GO terms (left side) enriched by genes they interacted with in the coexpression network.
