# Supplementary material for: Transcriptomic Analysis of Long Non-Coding RNA during Candida albicans Infection
Source: Genes (Basel). 2023 Jan 18;14(2):251. doi: 10.3390/genes14020251 (PMC9956080; doi:10.3390/genes14020251)
Supplement: Supplementary file 1 [file genes-14-00251-s001.zip › genes-2036761-supplementary.pdf]

Supplementary Material

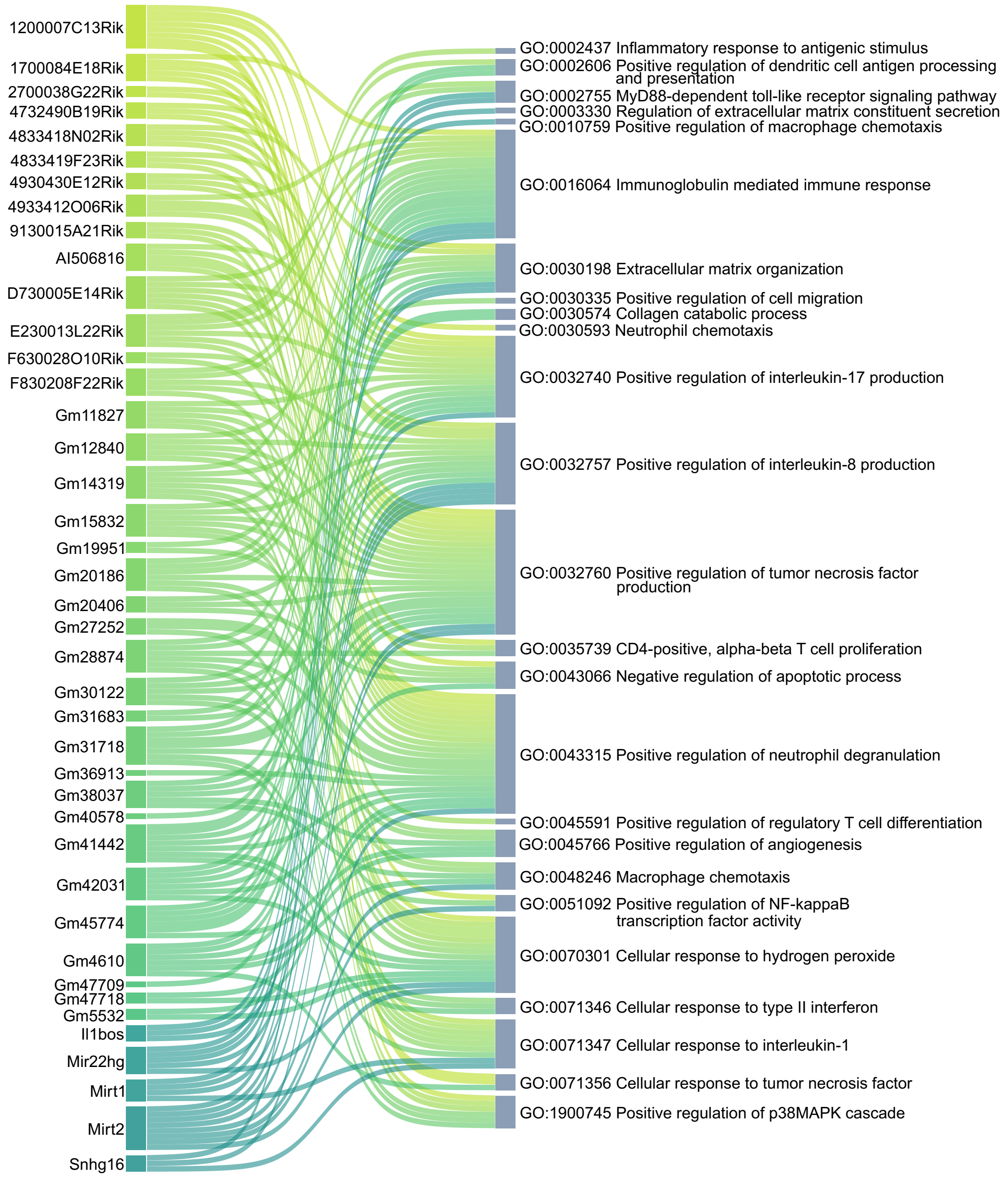

Figure S1: Sankey plot representing each lncRNA and the GO terms related to the genes it interacted with in the coexpression network.
